# “Textural analysis of multiparametric MRI detects transition zone prostate cancer”

**DOI:** 10.1007/s00330-016-4579-9

**Published:** 2016-09-12

**Authors:** Harbir S. Sidhu, Salvatore Benigno, Balaji Ganeshan, Nikos Dikaios, Edward W. Johnston, Clare Allen, Alex Kirkham, Ashley M. Groves, Hashim U. Ahmed, Mark Emberton, Stuart A. Taylor, Steve Halligan, Shonit Punwani

**Affiliations:** 1 Centre for Medical Imaging, University College London, 3rd Floor East, 250 Euston Road, London, NW1 2BU UK; 2 University College London Hospitals NHS Foundation Trust, 235 Euston Road, London, NW1 2BU UK; 3 Institute of Nuclear Medicine, University College London, University College Hospital, 235 Euston Road, London, NW1 2BU UK; 4 Research Department of Urology, University College London, 3rd Floor, Charles Bell House 67 Riding House Street, London, W1P 7NN UK; 5 Centre for Medical Imaging, University College London and University College London Hospitals NIHR Biomedical Research Centre, 250 Euston Road, London, NW1 2BU UK

**Keywords:** Magnetic Resonance Imaging, Prostate, Cancer, Diagnosis, Image Processing

## Abstract

**Objectives:**

To evaluate multiparametric-MRI (mpMRI) derived histogram textural-analysis parameters for detection of transition zone (TZ) prostatic tumour.

**Methods:**

Sixty-seven consecutive men with suspected prostate cancer underwent 1.5T mpMRI prior to template-mapping-biopsy (TPM). Twenty-six men had ‘significant’ TZ tumour. Two radiologists in consensus matched TPM to the single axial slice best depicting tumour, or largest TZ diameter for those with benign histology, to define single-slice whole TZ-regions-of-interest (ROIs). Textural-parameter differences between single-slice whole TZ-ROI containing significant tumour versus benign/insignificant tumour were analysed using Mann Whitney U test. Diagnostic accuracy was assessed by receiver operating characteristic area under curve (ROC-AUC) analysis cross-validated with leave-one-out (LOO) analysis.

**Results:**

ADC kurtosis was significantly lower (p < 0.001) in TZ containing significant tumour with ROC-AUC 0.80 (LOO-AUC 0.78); the difference became non-significant following exclusion of significant tumour from single-slice whole TZ-ROI (p = 0.23). T1-entropy was significantly lower (p = 0.004) in TZ containing significant tumour with ROC-AUC 0.70 (LOO-AUC 0.66) and was unaffected by excluding significant tumour from TZ-ROI (p = 0.004). Combining these parameters yielded ROC-AUC 0.86 (LOO-AUC 0.83).

**Conclusion:**

Textural features of the whole prostate TZ can discriminate significant prostatic cancer through reduced kurtosis of the ADC-histogram where significant tumour is included in TZ-ROI and reduced T1 entropy independent of tumour inclusion.

***Key Points*:**

• *MR textural features of prostate transition zone may discriminate significant prostatic cancer.*

• *Transition zone (TZ) containing significant tumour demonstrates a less peaked ADC histogram.*

• *TZ containing significant tumour reveals higher post-contrast T1-weighted homogeneity.*

• *The utility of MR texture analysis in prostate cancer merits further investigation.*

## Introduction

Management of early prostate cancer has been revolutionised by the use of multi-parametric MRI (mpMRI; using T2 and T1 weighted, diffusion-weighted and contrast- enhanced imaging) [1]. Nonetheless, transition zone (TZ) tumours remain more difficult to appreciate on mpMRI studies [2]; with reported sensitivity/specificity for detection of 0.53/0.83 compared with 0.80/0.97, respectively, for peripheral zone (PZ) tumours [1]. TZ tumour signal homogeneity has been advocated as a discriminator of significant grade disease at mpMRI and has been incorporated into the recently revised ‘Pi-RADS 2’ guidelines [3]; endorsed by the European Society of Urogenital Radiology and the American College of Radiology.

Textural analysis is an image-processing technique that can assess image signal heterogeneity (both at and beyond that appreciated by the human eye) by quantifying the coarseness and regularity of the spatial distribution of pixel grey level values within normal and pathological tissue. Macroscopic heterogeneity in medical images may reflect microscopic heterogeneity at the histopathological level, particularly in oncological imaging with recent demonstrations of utility in tumour detection/grading, prognosis and treatment response [4, 5]. Compared to CT, MRI offers the advantages of improved soft tissue contrast resolution and of a wealth of imaging data afforded by a multi-parametric approach. Recent studies have used MR textural analysis (MRTA) for lesion detection, classification, treatment response-evaluation and prediction for example in breast, brain, and rectal cancer [6–8]. A number of approaches to texture analysis exist, with one approach being quantification of features through histogram analysis [9].

It is possible the additional tissue heterogeneity data provided by textural analysis could augment the diagnostic accuracy of radiologists in detecting TZ tumours, where lesions tend to be subtle and difficult to differentiate from adjacent benign nodular tissue. In this study, image analysis was performed on a whole TZ basis with the aim of obviating the need for radiological pre-identification of tumour, thus increasing potential utility in computer-aided diagnosis (CAD) and to minimise inherent difficulties of small lesion contouring. The purpose of this study was to evaluate multiparametric MRI (mpMRI) derived histogram textural analysis parameters [9] for detection of transition zone (TZ) prostatic tumour.

## Materials and methods

Our local institutional review board approved the study and waived the requirement for individual consent for this retrospective study of consecutive patient data acquired as part of routine clinical care (R&D No: 12/0195).

### Patient cohort

The cohort comprised men with clinically suspected prostate cancer undergoing prostatic mpMRI prior to ‘20 zone’ template prostate mapping (TPM) biopsies (within 12 months) between 1 January 2010 to 31 December 2012 (*n* = 210). Men who had a peripheral zone tumour, undergone biopsy within 6 months prior to mpMRI, received previous treatment for prostate cancer, had metallic hip prostheses, or had incomplete mpMRI and/or TPM data sets were excluded (*n* = 143). In total sixty-seven men with mean age 63.4 years (45–80 years), mean PSA 9.2 ng/ml (0.2–39.0 ng/ml) and mean gland volume of 42.9 ml (15–101 ml) were accrued. Of these 29/67 (43 %) had no cancer, 26/67 (39 %) had ‘significant’ TZ cancer and 12/67 (18 %) had ‘insignificant’ TZ cancer (see below). Table 1 summarizes cohort demographics.

**Table 1:** Summary of demographic and ROI areas for recruited patients categorised by benign/insignificant transition (TZ) pathology and significant TZ tumours (MCCL = maximum cancer core length, G = Gleason grade, PSA = prostate specific antigen serum concentration, TPM = template mapping biopsy)

Cancer significance	Number (%)	Mean Age years (range)	Mean PSA ng/dL (range)	Mean prostate volume ml (range)	Median time interval mpMRI to TPM days (range)	Mean area TZ ROI cm^2^ (Total; range)	Mean area TZ tumour cm^2^ (Total; range)	Percentage TZ tumour area/TZ area (Total; range)
Benign OR	41	64	8.6	47.0	54	40.0	0.76	2.4
Insignificant (<4 mm MCCL AND ≤ G3 + 3)	(61 %)	64 (45-79)	(0.2-39)	(20-101)	(6-214)	(*n* = 41; 10.0-68.9)	(*n* = 12; 0.52-1.22)	(*n* = 12; 1.1-3.7)
Significant	26	63	9.9	37.2	56	29.9	1.45	5.3
(≥4 mm MCCL OR ≥ G3 + 4)	(39 %)	(52-80)	(0.3-35)	(15-78)	(16-145)	(*n* = 26; 9.3-61.2)	(*n* = 26; 0.65-3.80)	(*n* = 26; 1.7- 14.0)

### Multi-parametric magnetic resonance imaging

**Fig. 1:**
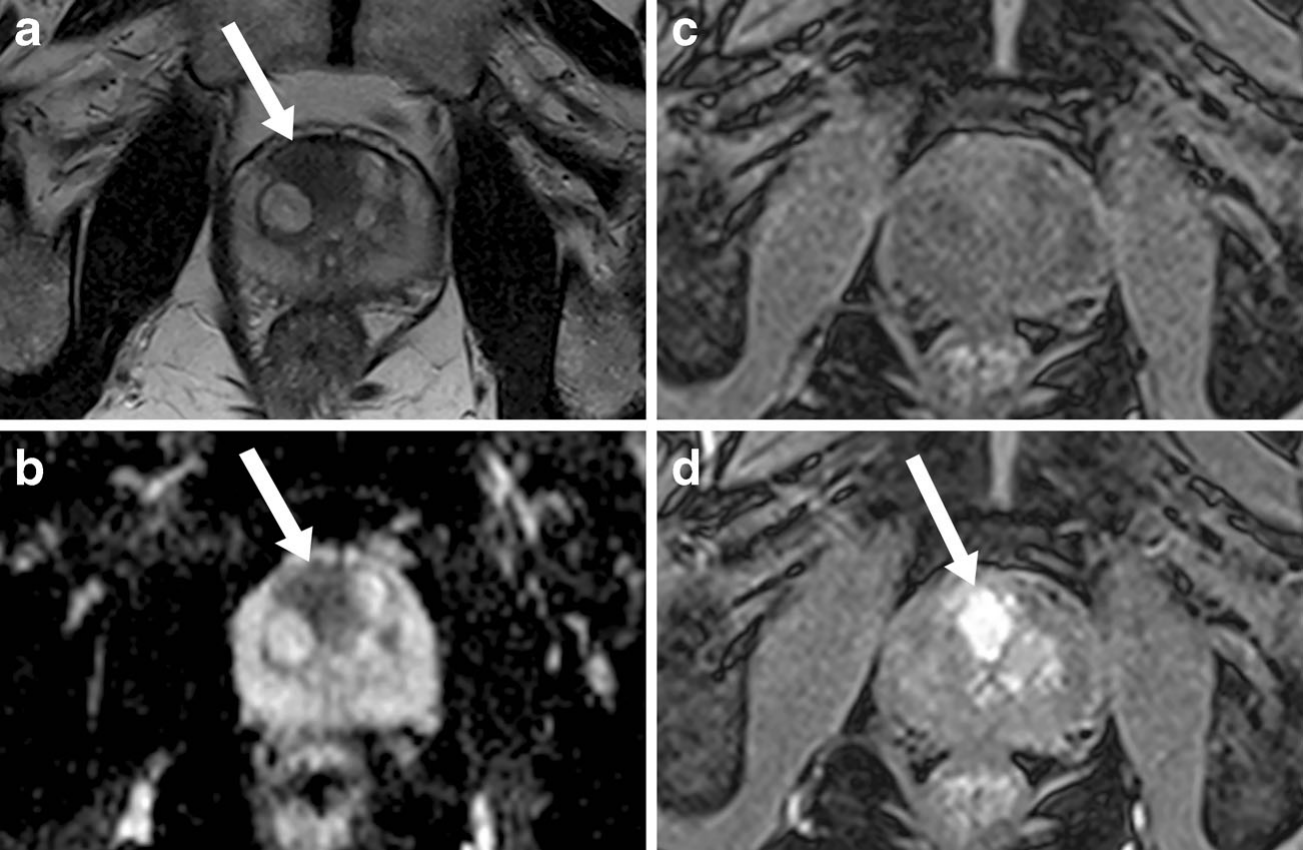
Demonstrates single slice axial images of significant tumours (arrow): (a) T2 weighted; (b) ADC map; (c) pre-contrast T1 weighted; and (d) early post contrast T1 weighted images in a 74-year-old patient with an anterior right transition zone tumour (Gleason 3 + 4; maximum cancer core length 11 mm) prior to transition zone contouring and histogram analysis

Subjects underwent 1.5T magnet mpMRI (Avanto, Siemens, Erlangen, Germany) with pelvic-phased array coil, following intravenous spasmolytic (Buscopan; Boehringer Ingelheim, Germany) 0.2 mg/kg (maximum 20 mg) to minimize bowel peristalsis. Full mpMRI parameters are given in Table 2. Figure 1 shows an example mpMRI with a significant TZ tumour.

**Table 2:** Multi-parametric MRI sequence parameters used for study; *dynamic contrast enhanced MRI – 0.2 ml/kg intravenous gadolinium contrast agent injected at the beginning of acquisition 6 at 3 ml/s followed by a saline flush of 20 ml; T2w TSE – T2 weighted turbo spin echo; EPI-DWI – echo planar imaging - diffusion weighted imaging; FLASH – fast low angle shot. Note coronal T2 acquisition was not used for image analysis (though remains part of the clinical scan). Apparent diffusion co-efficient (ADC) map automatically generated from the four b-values (mono-exponential). ‘Early post contrast T1’ image refers to the second image temporally from the point at which contrast first appears in the prostate gland (imaging every 16 seconds)

	T2w TSE axial/coronal	EPI DWI	T1 3D FLASH*
Repetition time (ms)	5170 / 5240	2100	5.61
Echo time (ms)	92 / 104	98	2.5
Flip angle (degrees)	180 / 150	90	15
Echo train length	22 / 24	172	n/a
Bandwidth (Hz/Px)	190 / 190	968	300
Field of view (mm)	180 / 180	260	260
Phase FoV %	100 / 100	100	100
Slice thickness (mm)	3 / 3	5	3
Slice gap (mm)	0.3 / 0.3	0	0.6
Averages	2 / 2	16	1
Phase encoding direction	A > P / R > L	A > p	A > P
Fat saturation	No / No	Yes	Yes
Base matrix	256 / 256	172	192
Matrix phase %	95 / 95	100	100
b-values (s.mm^-2^)	n/a	0, 300, 500, 1000	n/a
Number of acquisitions	1 / 1	1	35
Temporal resolution (s)	n/a	n/a	16
Total acquisition time (min)	3m54s / 4m18s	3m39s	10 m

### Transperineal template-prostate-mapping biopsy

TPM followed mpMRI with a median interval of 56 days (2 to 214 days); method as previously described [10, 11]. In brief, systematic biopsy of the whole gland was performed through a brachytherapy template-grid and 5-mm sampling frame, giving a uniform sampling density of approximately 1 core/cc of prostatic tissue. Biopsies were grouped and potted into 20 zones, modified from the technique reported by Barzell et al. [12].

### Histopathology review

Subjects were grouped according to a previously used definition of cancer significance [13] whereby TPM maximum cancer core length (MCCL) values were used to infer volume, following demonstration that a TPM MCCL of ≥4 mm approximates to a tumour volume of ≥0.2 ml [14].

Clinically significant disease was defined as ≥ Gleason 3 + 4 OR ≥4 mm MCCL, while other disease (i.e., ‘low risk’ <4 mm MCCL AND ≤ Gleason 3 + 3) was classified as non-significant and grouped with patients demonstrating benign TZ histology [15].

### Histology-MRI Matching

Two radiologists in consensus (HSS, SP; with 8 years and 4 years of experience, respectively, for mpMRI interpretation), aware of histopathological findings, reviewed each dataset using Osirix (version 3.5.1; Geneva, Switzerland) and matched the single axial slice depicting the TZ focus most suspicious for disease to location of disease confirmed according to histopathology. If no tumour was present, the radiologists selected the slice with the largest TZ anteroposterior diameter. Where biopsy-positive significant tumour was present, the radiologists also contoured significant tumours on each ADC, T2 and T1 weighted TZ single slice image to analyse the effect of initially including and then excluding significant tumours from the single-slice whole TZ ROI drawn by a third radiologist (below). TZ tumour contoured on T2 images was used for tumour area estimation (relative to total TZ area).

A third radiologist (SB; with 2 years of experience for mpMRI interpretation), blinded to biopsy data and earlier tumour region of interest (ROI) placement, contoured solely the entire TZ on the selected slice, on matched ADC, T2 and early post-contrast T1 weighted images for each patient for subsequent MRTA.

The percentage of TZ replaced by tumour was quantified by [(tumour area / TZ area)*100].

### MR Textural Analysis (MRTA)

The ADC map, T2 and early post-contrast T1 weighted segmented TZ regions from the selected slice (containing and initially including tumour where present) underwent MRTA (BG, with 9 years of experience in texture analysis) using proprietary TexRAD research software (version 3.3, TexRAD Ltd, Feedback Plc, Cambridge, UK) with automated texture parameter extraction. A subsequent separate analysis examined single-slice whole TZ ROIs excluding significant tumours to assess the effect on textural parameters.

MRTA comprised image histogram analysis to quantify first-order statistics of entropy, skewness, and kurtosis of the TZ ROIs. These parameters reflect, to varying degrees, the number, intensity and variability of areas of high and low signal intensity within the TZ [9]. Absolute T2 and T1 weighted signal intensities are not comparable across patients without standardisation, and; therefore, T1 and T2 mean pixel, mean positive pixel and standard deviation values were not analysed further (unlike mean ADC). By contrast, measurement of entropy, kurtosis and skewness rely on the shape of the histogram, i.e., relationships between pixel intensities and not on the absolute pixel intensity values.

### Statistical Analysis

For each individual textural variable, the significance of difference between non-significant/benign TZ and significant tumours containing TZ were assessed using the two tailed Mann Whitney U test (statistical significance assigned at *p* < 0.05). These analyses were repeated after exclusion of significant tumour area from the single-slice whole TZ ROI to determine if the observed differences in textural parameters could be directly attributed to inclusion of significant tumour within the analyzed TZ ROI. Receiver-operating characteristics (ROC) analyses characterised the performance of TZ textural features extracted from each of ADC, T1 and T2 weighted TZ ROI to predict significant TZ prostate cancer.

The area under the ROC curve (ROC AUC) and 95 % confidence intervals for each parameter identified the best performing individual parameters and in combination using multivariate ROC-AUC analysis [16]. Leave-one-out (LOO) analysis [17] validated classification performance for best performing univariate and combined parameters.

All statistical analyses were performed using SPSS statistics for Windows (version 16; IBM, Armonk, NY) and MedCalc for Windows (version 9.2.0.0; MedCalc software, Mariakerke, Belgium).

## Results

### Patient cohort

Mean tumour area was 1.45 cm^2^ (0.65 to 3.8) for patients with significant tumour at histology. Mean TZ area was 42.4 cm^2^ (10.0 to 68.9) for patients with benign histology; 33.9 cm^2^ (18.7 to 58.8) for patients with non-significant cancer, and 29.9 cm^2^ (9.3 to 61.2) for patients with significant cancer. Mean proportion of TZ replaced by tumour was 5.3 % (range 1.7 to 14.0 %) for patients with significant tumour (Table 1). Table 3 denotes the median values and interquartile ranges of whole TZ MR textural parameters for patients with non-significant/benign and for patients with significant tumour both including and excluding the significant tumour. P-values denote the statistical significance of differences between whole TZ for patients with non-significant/benign histology and patients with significant tumour for TZ ROIs (again including and excluding the significant tumour). The ROC-AUC for whole TZ textural parameters for each of the three MRI sequences, for classification of a TZ image as containing significant tumour, are also detailed.

**Table 3:** Median values (and interquartile range 25 %-75 %) for ADC map, early post-contrast T1, and T2 weighted TZ ROIs for kurtosis, entropy and skewness derived from first order histogram analysis both including and excluding significant tumour where present. P-values have been calculated using two-tailed Mann Whitney U test. Those values in bold indicate most significant difference in values (i.e., between non-significant/benign and significant tumours TZ) for each sequence where applicable. Right hand column compares receiver operator characteristics (ROC) area under curve values for differentiating TZ ROIs containing significant TZ tumours from non-significant/benign histology for the MRTA parameters (values in parentheses indicate 95 % confidence intervals)

Cohort/ Sequence	Non-significant/ benign TZ median (interquartile range)	Significant cancer TZ ROI includes tumour median (interquartile range*)*	Significant cancer TZ ROI excludes tumour median (interquartile range)	p-value whole TZ ROI includes significant tumour	p-value whole TZ ROI excludes significant tumour	ROC area under curve (95 % CI)
Kurtosis						
ADC	0.09 (-0.14 to 0.59)	-0.51 (-0.69 to 0.01)	0.10 (-0.31 to 0.45)	<0.001	0.23	0.80 (0.69,0.91)
Early T1	-0.12 (-0.34 to 0.26)	-0.05 (-0.56 to 0.40)	0.06 (-0.47 to 0.37)	0.96	0.84	0.50 (0.35,0.65)
T2	1.51 (0.65 to 2.73)	1.18 (0.40 to 2.86)	1.00 (0.33 to 2.50)	0.78	0.35	0.52 (0.37,0.67)
Entropy						
ADC	5.90 (5.68 to 6.06)	5.67 (5.42 to 5.83)	5.57 (5.38 to 5.91)	0.005	<0.001	0.69 (0.58,0.84)
Early T1	5.51 (5.41 to 5.62)	5.37 (5.09-5.54)	5.32 (5.12 to 5.53)	0.004	0.004	0.70 (0.57,0.84)
T2	5.12 (4.94 to 5.24)	5.04 (4.82 to 5.14)	5.02 (4.90 to 5.18)	0.13	0.34	0.61 (0.47,0.75)
Skewness						
ADC	-0.04 (-0.31 to 0.20)	0.00 (-0.13 to 0.25)	-0.12 (-0.44 to 0.05)	0.31	0.27	0.58 (0.44,0.71)
Early T1	0.16 (-0.24 to 0.31)	0.08 (-0.08 to 0.45)	0.25 (0.01 to 0.46)	0.49	0.06	0.55 (0.41,0.70)
T2	0.60 (0.30 to 1.00)	0.74 (0.57 to 1.03)	0.72 (0.50 to 1.13)	0.35	0.47	0.57 (0.43,0.71)
Mean						
ADC	0.99 (0.88 to 1.00)	0.80 (0.77 to 1.00)	0.92 (0.80 to 1.05)	0.004	0.13	0.71 (0.58,0.85)
Bivariate Model						
ADC Kurtosis + Early T1 Entropy						0.86 (0.77,0.95)

### TZ textural metrics

All analyses were performed on a whole TZ basis (initially incorporating significant tumour where present). Mean and median TZ ADC were significantly higher (0.99 and 0.97 x 10-3 mm2/s, respectively, range 0.67 to 1.77) for patients with non-significant/benign histology than those with significant tumour (0.79 and 0.77 x 10-3 mm2/s respectively, range 0.07 to 1.22) (*p* = 0.004).

#### Kurtosis

Median ADC, T1 and T2 kurtosis were 0.09, -0.12 and 1.51, respectively, for non-significant/benign histology versus -0.51, -0.05 and 1.18, respectively, for TZ containing significant tumour. Median ADC kurtosis was significantly lower for patients with significant tumour (*p* < 0.001); whereas median T1 and T2 kurtosis were not significantly different between the groups (*p* = 0.96 and 0.78, respectively).

#### Entropy

Median ADC, T1 and T2 entropy were 5.90, 5.51 and 5.12, respectively, for non-significant/benign histology and 5.67, 5.37 and 5.04, respectively, for TZ containing significant tumour. Median ADC and T1 entropy were lower for patients with significant tumour (*p* = 0.005 and 0.004, respectively) whilst T2 entropy did not reach significance (*p* = 0.13).

#### Skewness

Median ADC, T1 and T2 skewness were -0.04, 0.16 and 0.60, respectively, for non-significant/benign histology versus 0.00, 0.08 and 0.74, respectively, for TZ containing significant tumours. There were no significant differences between the two groups (*p* = 0.31 to 0.49).

### Effect of excluding significant tumours on whole TZ textural metrics

Following exclusion of significant tumours from the single-slice whole TZ ROI, there was no longer a significant difference between median ADC value (*p* = 0.13), when patients with those significant tumours were compared with those with non-significant/benign histology.

#### Kurtosis

Following exclusion of significant tumours from the single-slice whole TZ ROI, there was no longer a significant difference between median ADC kurtosis, when patients with significant tumours were compared with those with non-significant/benign histology (*p* = 0.23). Median T1 and T2 kurtosis differences remained non-significant (*p* = 0.84 and 0.35, respectively; Table 3).

#### Entropy

Significant differences between T1 and ADC entropy remained, when patients with significant tumours and those with non-significant/benign histology were compared following exclusion of significant tumours from the former ROI (*p* <0.01, Table 3).

#### Skewness

Median ADC, T1 and T2 skewness did not demonstrate any consistent difference between patients with significant tumours and those with non-significant/benign histology following exclusion of significant tumours from the TZ ROI.

**Fig. 2:**
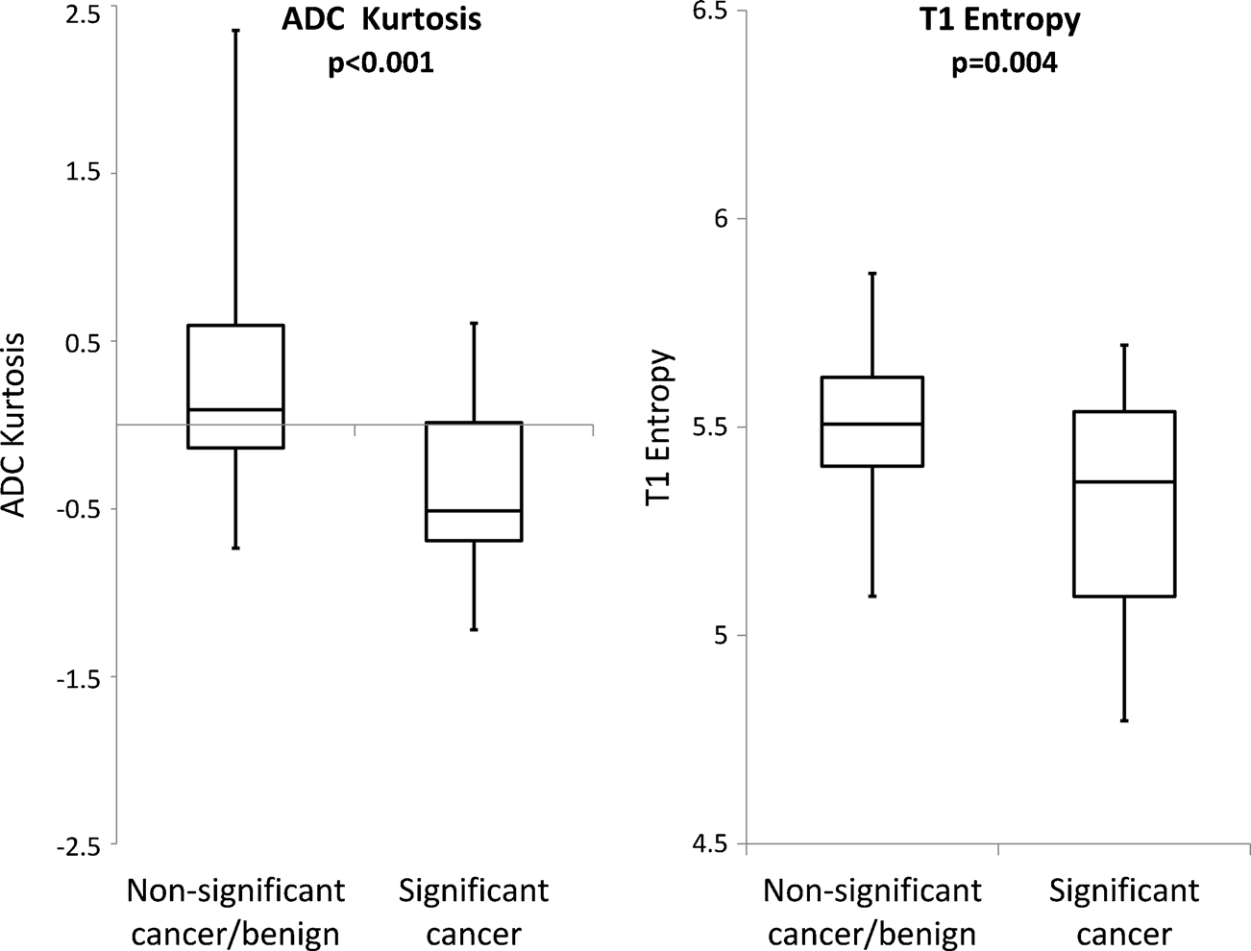
Box plots showing best performing textural discriminators of TZ ROIs containing significance and non-significance using ADC kurtosis and early post-contrast T1. In each box plot the box indicates interquartile range; line indicates median and whiskers indicate most deviated data points/range. Two tailed Mann Whitney U p-values are also given for each parameter

Box-and-whiskers plot of the best performing textural parameters are illustrated in Fig. 2.

### Disease classification by univariate textural metrics

The best performing classifier for ADC was kurtosis (0.80; 95 % CI 0.69 to 0.91). Entropy yielded the highest AUC for early T1 post-contrast image (0.70; 95 % CI 0.57 to 0.84). This analysis did not show significant differences on T2 weighted whole TZ textural analysis with the best performing T2 parameter being T2 entropy (AUC of 0.61; 95 % CI 0.47 to 0.75). LOO validation demonstrated ROC-AUC 0.78 (95 % CI 0.66 to 0.90) for ADC kurtosis and 0.66 (95 % CI 0.52 to 0.80) for T1 entropy.

### Disease classification by multivariate textural metrics

**Fig. 3:**
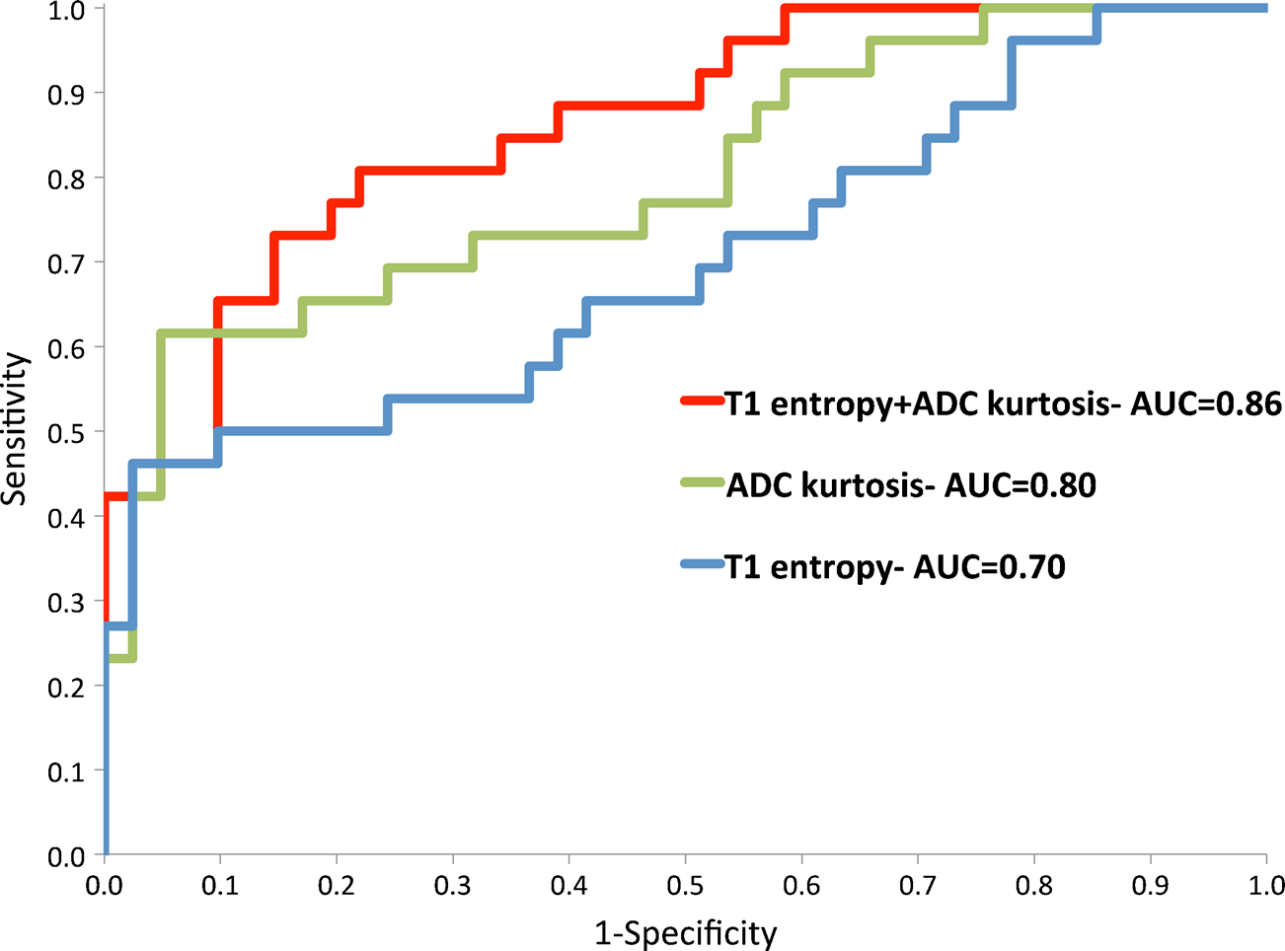
Receiver operating characteristic (ROC) curves of the two best performing textural features and bivariate combination for discrimination of transition zone ROIs containing significant prostatic tumours from non-significant TZ with area under curve (AUC) values as shown

**Fig. 4:**
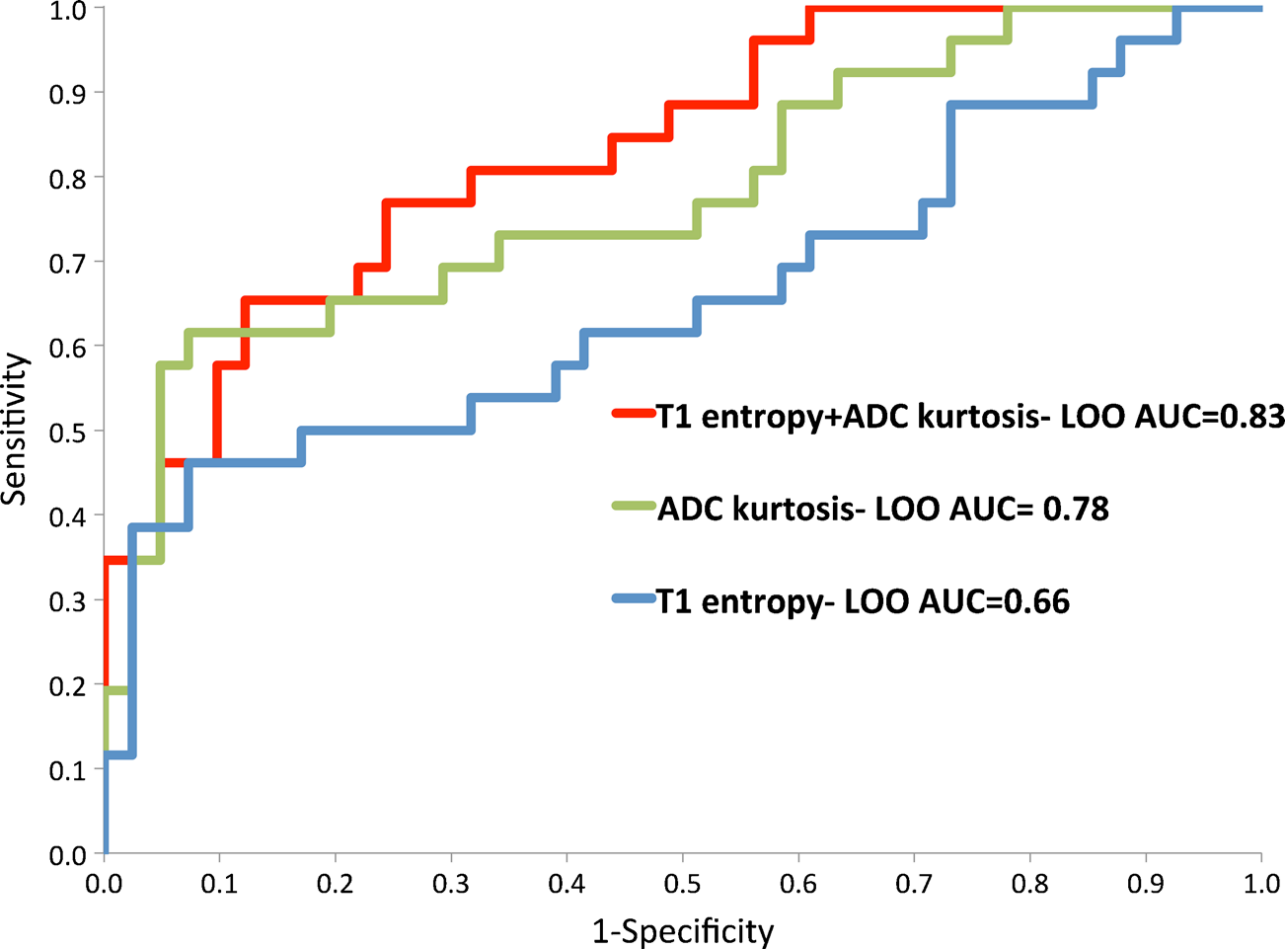
Receiver operating characteristic (ROC) curves of the two best performing textural features and bivariate combination for discrimination of TZ ROIs containing significant prostatic tumours from non-significant TZ ROIs after leave-one-out (LOO) analysis with area under curve (AUC) values as shown

ROC-AUC for the best performing significant univariate parameters and bivariate combination of these parameters are shown in Fig. 3. The two best performing parameters for ADC (kurtosis) and T1 (entropy) combined in the bivariate model gave ROC-AUC of 0.86 (95 % CI 0.77 to 0.95). LOO analysis of this bivariate model yielded ROC-AUC 0.83 (95 % CI 0.74 to 0.93) and is shown in Fig. 4.

## Discussion

This study evaluated the diagnostic accuracy of textural parameters, derived from clinical prostate mpMRI, for detection of TZ cancer. Previous work has confirmed that quantitative mpMRI parameters (e.g., ADC) can differ between benign and cancerous TZ regions [18]. Unlike previous studies, here we derived single-slice whole TZ textural parameters (including cancer pixels where cancer was present) and evaluated differences in the histographic pixel distribution between patients with and without significant cancer.

We found that textural features from an image of the entire TZ are altered significantly, when containing even a small proportion of significant cancer, which no longer holds true (for best performing textural parameter) when the same tumour is excluded from the analysis. Overall, classification of TZ tumour-containing slices by best performing single textural parameter and/or bivariate combination (ROC-AUC 0.80 to 0.86) was comparable with previously reported visual detection of TZ tumour by radiologists (ROC-AUC 0.73 to 0.84) [19, 20].

Kurtosis is a measure of histogram “peakedness”. Positive kurtosis indicates a more peaked distribution of pixel signal intensities. We found reduced ADC kurtosis was the best univariate classifying textural feature (ROC-AUC 0.80 on ADC images) on a whole TZ basis and demonstrated a higher ROC-AUC than the non-textural parameter of ADC mean (ROC-AUC 0.71 on ADC images). A larger cohort is required to test the statistical significance of this difference. We expect textural measures, based on the relationship of pixels in a given ROI rather than absolute pixel intensities, are likely more robust to variations between individuals and scanners. Whilst kurtosis measures from T2 and early post-contrast T1 weighted images performed less well for the detection of cancer. In spite of the relatively modest ratio of significant tumour compared to remaining TZ area (~5 %), we have found that exclusion of this radiologically visible tumour from the single-slice whole TZ ROI results in loss of significance in the difference in ADC kurtosis between the two groups. We posit this to be the result of elimination of a second differing signal intensity ‘population’ (i.e., a low ADC significant tumour) from the TZ ROI resulting in normalisation of kurtosis when compared to the non-significant/benign cohort reflected in more ‘peaked’ values. Wibner et al. [21] have previously demonstrated the utility of ADC-derived textural parameters in TZ tumour detection and differentiating grade based on lesion analysis. The textural analysis approach may obviate previously described efforts to normalise by using ADC ratios (normalized to non-tumorous tissues) [22], perform whole lesion measurement and/or generate histogram analysis including median and low percentile ADC values [23].

Entropy is a measure of image ‘busyness/irregularity’ and, as such, the observation of significantly lower mean TZ entropy values in patients with significant cancer would reflect increased overall signal homogeneity. Post-contrast T1 weighted image entropy was the second best univariate classifier, in keeping with previously observed homogenous enhancement features of TZ tumours [20]. However, the observation that the significant difference in T1 entropy between TZ containing significant tumours and non-significant/benign TZ persists even after the exclusion of radiologically visible tumours in the former group reflected more homogeneous enhancement throughout the whole TZ in these cases. The mechanism for this is unclear, though growing evidence implicates chronic inflammation as a contributor to prostate cancer development and progression [24] and present in benign prostate tissue associated with high-grade prostate cancer [25]. The finding of more homogenous enhancement in the ‘non-malignant’ parts of TZ harbouring significant tumours may reflect these aspects of prostate cancer ontogeny. ADC entropy was also significantly lower in single-slice TZ ROIs containing tumours and this relationship persisted following removal of cancer from the TZ ROI possibly due to the same/similar mechanism. Additionally, at the time these data were acquired, as demonstrated in Table 2, the lowest b-value used for calculation of the ADC map was 0 sec/mm2, and it is possible the findings of ADC entropy may at least in part reflect a perfusion difference component. These findings may not have been appreciated in prior quantitative imaging studies, most of which concentrate on assessing the vascular properties of tumours themselves, and further confirmatory (histologically correlative) studies are required.

Several T2 weighted features aid visual identification of TZ tumours; including homogeneously reduced lesion signal intensity, ill-defined lesion margins and lenticular lesion shape [19]. However, on a whole TZ basis, the current study does not confirm that quantitatively reduced whole TZ entropy (increased homogeneity) on T2 weighted images is a particularly good discriminator. This may relate to the relatively small area of tumours compared with background non-tumorous TZ and/or relate to the study size. Additionally, ADC and early post-contrast T1 images reflect processes occurring on a ‘microscopic’ level and can reflect field changes (for example, subtle inflammatory change) that may explain the significance of differences between benign TZ and that harbouring significant tumours. Whilst several studies have described homogenous reduced tumoral T2 signal compared to benign TZ, the remaining (and predominant approximately 95 % by area) ‘normal’ TZ glandular tissue is variably heterogeneous across patients and images are more reflective of macroscopic differences and subtle changes in T2 signal induced by background inflammation may be masked.

Skewness is a measure of histogram asymmetry; a zero value indicates a symmetrical distribution around the mean. Skewness was a relatively poor classifier of TZ cancer when applied to any image sequence. It has been speculated [23] that in prostate tumours densely packed with malignant cells, the resulting histograms from tumour ROIs are likely to be less skewed compared with tumours with more heterogeneous cellular density. However, we evaluated skewness for all pixels within the whole TZ, and the proportion of tumour pixels may have been insufficient to influence skewness significantly particularly given such variation in cellular density is not captured by traditional histological grading [26].

To our knowledge, this study is distinct from other work as it assessed in vivo textural features derived from each mpMRI sequence for the entire TZ. The study was conducted as a proof of concept for the purpose of detecting significant TZ tumours. Future work will examine the translation of this concept over whole TZ volume to examine utility in augmenting visual radiologist assessment (e.g., added value of prompting readers to areas for examination which may contain significant cancer) and/or automated detection. Interpretation of images by radiologists is complex [27] and, although an assessment of texture is made, to date this visual textural assessment has not been characterised objectively. We believe that textural analysis of each multiparametric image is analogous to the manner by which radiologists visually localise TZ cancer on mpMRI [4]. Other workers [e.g., 28, 29] have examined first and second order (e.g., two-dimensional grey-level co-occurrence matrix) textural features from one or more mpMRI sequences from ROIs drawn around individual TZ tumours. Such approaches, while informative, have less clinical application, since they are more computationally intensive and require the radiologist to first identify areas of concern. Furthermore, where textural features of small lesions are evaluated at larger spatial scales, these features can be biased significantly by region boundary. In contrast, assessment of the entire TZ texture requires less development as a clinical diagnostic tool (i.e., only segmentation of the TZ from the PZ, which may be an automated/semi-automated procedure [30]) and minimizes boundary-related effects.

Our study has some limitations. All mpMRI images were acquired on a 1.5 T scanner and generalizability to 3 T platforms was not assessed. However, our 1.5 T mpMRI protocol was in keeping with recommendations from a European Consensus meeting [31]. We did not use endorectal coils, and note that the European Consensus Meeting failed to agree regarding their necessity even at 1.5 T. We routinely perform DCE MRI using a higher spatial resolution and lower temporal resolution than some other centres as advocated by recent guidelines [3, 32]. Therefore, we expect the generalizability of post-contrast T1 weighted textural feature findings to be limited to centres that similarly maintain higher spatial resolution. We did not have access to radical prostatectomy specimens as a reference ground-truth. However, employment of a TPM based reference standard [33], which is a significant improvement over conventional TRUS biopsy [14], can help avoid the spectrum bias towards more severe cases that occurs with studies when prostatectomy specimens are mandated. We acknowledge that there could be some error when registering TPM histopathology to mpMRI. However, it should be noted that prostatectomy specimens themselves are not free from registration errors induced by processing (shrinkage, distortion, and tissue-loss) [34]. Lastly, we manually contoured the single-slice TZ ROIs though we do not expect this to unduly affect the results and envisage automated segmentation could be used as available.

Our results have immediate clinical relevance, and confirm textural features may inform CAD software to highlight images on which significant tumours could be present within the TZ. In developing this whole TZ approach using MRTA as an imaging biomarker, it is recognised that a series of studies would be required to further validate the findings as per various ‘roadmap’ approaches in contemporaneous guidelines (http://www.cancerresearchuk.org/sites/default/files/imaging_biomarker_roadmap_for_cancer_studies.pdf). This approach could be adapted to several scenarios, subject to demonstration of efficacy, for example, by drawing attention to slice(s), which may require further examination for radiologists, probability of significant tumours being present across entire TZ and predictive information if there are relevant ‘field’ changes across the TZ. The United Kingdom NICE guidelines have adopted mpMRI for detection of prostate cancer in patients with a negative non-targeted TRUS biopsy (http://www.nice.org.uk/guidance/cg175/chapter/recommendations). Our textural evaluation technique may have particular relevance for such patients who are more likely to have TZ tumours that are systematically undersampled by TRUS [34].

## Abbreviations

MCCL: Maximum cancer core length (mm)
mpMRI: Multi-parametric MRI
MRTA: MRI textural analysis
TPM: Transperineal template prostate mapping biopsy
TZ: Transition zone of the prostate
